# Impact of the COVID-19 pandemic on non-COVID-19 hospital mortality in patients with schizophrenia: a nationwide population-based cohort study

**DOI:** 10.1038/s41380-022-01803-4

**Published:** 2022-10-07

**Authors:** Laurent Boyer, Guillaume Fond, Vanessa Pauly, Veronica Orléans, Pascal Auquier, Marco Solmi, Christoph U. Correll, Dong Keon Yon, Pierre-Michel Llorca, Karine Baumstarck-Barrau, Antoine Duclos

**Affiliations:** 1 CEReSS - Health Service Research and Quality of Life Center, EA3279, Aix-Marseille University, Marseille, 13005 France; 2 Fondation FondaMental, F-94010 Créteil, France; 3 Department of Psychiatry, University of Ottawa, Ottawa, ON Canada; 4 Department of Mental Health, The Ottawa Hospital, Ottawa, ON Canada; 5 Ottawa Hospital Research Institute (OHRI), Clinical Epidemiology Program, University of Ottawa, Ottawa, ON Canada; 6 School of Epidemiology and Public Health, Faculty of Medicine, University of Ottawa, Ottawa, ON Canada; 7 Department of Psychiatry, The Zucker Hillside Hospital, Glen Oaks, NY USA; 8 Department of Psychiatry and Molecular Medicine, The Donald and Barbara Zucker School of Medicine at Hofstra/Northwell, Hempstead, NY USA; 9 Charité-Universitätsmedizin Berlin, corporate member of Freie Universität Berlin, Humboldt-Universität zu Berlin, and Berlin Institute of Health, Department of Child and Adolescent Psychiatry, Berlin, Germany; 10 Department of Data Science, Sejong University College of Software Convergence, Seoul, Republic of Korea; 11 Department of Pediatrics, Kyung Hee University Medical Center, Kyung Hee University College of Medicine, Seoul, South Korea; 12 Center for Digital Health, Medical Science Research Institute, Kyung Hee University College of Medicine, Seoul, South Korea; 13 Department B of Psychiatry, Clermont-Ferrand Teaching Hospital, Clermont-Ferrand, 63000 France; 14 RESHAPE - Research on Healthcare Performance Lab, Inserm U1290, Claude Bernard Lyon 1 University, Lyon, 69424 France

**Keywords:** Schizophrenia, Addiction

## Abstract

It remains unknown to what degree resource prioritization toward SARS-CoV-2 (2019-nCoV) coronavirus (COVID-19) cases had disrupted usual acute care for non-COVID-19 patients, especially in the most vulnerable populations such as patients with schizophrenia. The objective was to establish whether the impact of the COVID-19 pandemic on non-COVID-19 hospital mortality and access to hospital care differed between patients with schizophrenia versus without severe mental disorder. We conducted a nationwide population-based cohort study of all non-COVID-19 acute hospitalizations in the pre-COVID-19 (March 1, 2019 through December 31, 2019) and COVID-19 (March 1, 2020 through December 31, 2020) periods in France. We divided the population into patients with schizophrenia and age/sex-matched patients without severe mental disorder (1:10). Using a difference-in-differences approach, we performed multivariate patient-level logistic regression models (adjusted odds ratio, aOR) with adjustment for complementary health insurance, smoking, alcohol and substance addiction, Charlson comorbidity score, origin of the patient, category of care, intensive care unit (ICU) care, major diagnosis groups and hospital characteristics. A total of 198,186 patients with schizophrenia were matched with 1,981,860 controls. The 90-day hospital mortality in patients with schizophrenia increased significantly more versus controls (aOR = 1.18; *p* < 0.001). This increased mortality was found for poisoning and injury (aOR = 1.26; *p* = 0.033), respiratory diseases (aOR = 1.19; *p* = 0.008) and for both surgery (aOR = 1.26; *p* = 0.008) and medical care settings (aOR = 1.16; *p* = 0.001). Significant changes in the case mix were noted with reduced admission in the ICU and for several somatic diseases including cancer, circulatory and digestive diseases and stroke for patients with schizophrenia compared to controls. These results suggest a greater deterioration in access to, effectiveness and safety of non-COVID-19 acute care in patients with schizophrenia compared to patients without severe mental disorders. These findings question hospitals’ resilience pertaining to patient safety and underline the importance of developing specific strategies for vulnerable patients in anticipation of future public health emergencies.

## Introduction

Over the past two years, patients with coronavirus disease 2019 (COVID-19) have overwhelmed hospital capacities due to the high volume of cases mobilizing most of the available resources in health care staff, beds and equipment [1, 2]. From March 2020 to December 2020, France experienced two significant COVID-19 waves (during spring and autumn) triggering nationwide lockdowns and the cancellation of planned care. Excessive workload and resource prioritization towards COVID-19 cases may have disrupted non-COVID-19 care and affected health outcomes [3].

Schizophrenia is a chronic and severe mental disorder affecting 20 million people worldwide [4]. Schizophrenia is associated with a substantial decrease in life expectancy of roughly 15 years as a result of eight- to almost ten-fold increase of accidents, suicide, non-natural mortality risk, and physical illnesses including seven-fold increased mortality for pneumonia, three- to four-fold-increase for infectious, endocrine, respiratory, urogenital, diabetes mortality, two- to three-fold increased risk of alcohol, gastrointestinal, renal, nervous system, cardio-cerebrovascular, natural-causes mortality, and 30% to almost 100% increased risk of liver, cerebrovascular, or any cancer mortality [5–8]. Compared with patients without a diagnosis of mental disorder, patients with schizophrenia face greater difficulties in accessing and receiving physical health care [9–11]. This gap in physical health care extends across different conditions, including but not limited to cardiovascular disease, with a meta-analysis including 47 studies conducted before COVID-19 pandemic reporting lower screening, catheterization or revascularization in coronary artery disease, intravenous thrombolysis for stroke, and treatment with any and with specific medications for CVD across all mental disorders and in particular in those with schizophrenia [12]. Recent works reported the existence of major disparities in health and health care for COVID-19 between patients with schizophrenia and patients without a diagnosis of mental disorder [13–17], including lower rates of COVID-19 vaccination [18]. It remains unknown to what degree excessive workload and resource prioritization toward COVID-19 care had affected access, quality and safety of non-COVID-19 care in patients with schizophrenia who are already affected by health care disparities [19].

In this study, we aimed to establish whether the impact of the COVID-19 pandemic on non-COVID-19 hospital mortality and access to hospital care differed between patients with schizophrenia and patients without a diagnosis of severe mental disorder. We hypothesized worse outcomes during the pandemic than before both in general population and in people schizophrenia, but that this worsening would be significantly more pronounced in people with schizophrenia.

## Methods

### Study design, sources and population

In this nationwide population-based cohort study, we used data from the Programme de Médicalisation des Systèmes d’Information (PMSI database), the French national hospital database in which administrative and medical data are systematically collected for acute (PMSI-MCO) and psychiatric (PMSI-PSY) care. The PMSI database is based on diagnosis-related groups with all diagnoses coded according to the 10th version of the International Classification of Diseases (ICD-10) and procedural codes from the Classification Commune des Actes Médicaux (CCAM). The PMSI database is used to determine financial resources and is frequently and thoroughly verified by both its producer and the paying party with possible financial and legal consequences [20]. Data from the PMSI database are anonymized and can be reused for research purposes. A unique anonymous identifier enables to link the different inpatient stays of the patients. The study was approved for ethical considerations by the French National Data Protection Commission (No. F20211214152715: https://www.health-data-hub.fr/projets/etude-en-vie-reelle-de-limpact-de-la-pandemie-covid-19-sur-lacces-et-la-qualite-de-la-prise). The manuscript follows the REporting of studies Conducted using Observational Routinely-collected health Data (RECORD) Statement [21].

We included all hospital admissions between March 1, 2019 and December 31, 2019 (pre-COVID-19 period) and between March 1, 2020 and December 31, 2020 (COVID-19 period) according to the following criteria: aged 18 years or older, admitted for acute care without COVID-19 (ICD-10 codes different from U071* in PMSI-MCO, this ICD-10 code U071* has been reported to be valid for detecting COVID-19 hospital stays [22]), and a length of hospital stay > 24 h (to exclude ambulatory care) except if the patients died within 24 h. We excluded patients with a severe mental disorder diagnosis other than schizophrenia: bipolar disorder or recurrent major depression (ICD-10 codes = F30* or F31* or F33*).

### Procedure

We defined two groups. The first group included patients who had a diagnosis of schizophrenia according to specific ICD-10 codes (i.e., F20*, F22*, or F25*). To control for downcoding, patients with schizophrenia were researched in either the acute or psychiatric PMSI databases from 2015 to 2020. The second group included patients who did not have a diagnosis of severe mental disorder according to the same procedure. Patients without a diagnosis of severe mental disorder were matched to patients with schizophrenia in a 10:1 ratio according to age (±5 years) and sex.

### Outcomes

The primary outcome was the difference in people with schizophrenia versus those without mental disorders in the pre- versus during COVID-19 periods in 90-day hospital non-COVID-19 mortality. Secondary outcomes were 30-day hospital mortality and patient case mix (i.e., admission-diagnosis distribution). Follow‐up for mortality was censored after hospital discharge. We performed analyses on both 30- and 90-day mortality to consider previous works suggesting that evaluation of hospital performance based on 30-day mortality may underestimate outcomes and therefore substantially misrepresent institutional performance compared with 90-day mortality [23–25].

### Collected data

We gathered the following patients’ sociodemographic (age classes: 18–24, 25–34, 35–44, 45–54, 55–64, 65–74, 75–84, 85–94 and ≥95 years; sex: male, female; complementary health insurance providing access to free health coverage for the bottom 10% of households [26]: yes, no) and clinical data [comorbidities (smoking addiction: yes, no; alcohol addiction: yes, no; substance addiction: yes, no; Charlson Comorbidity score based on ICD-10 codes: 0, 1–2, ≥3 [27]), characteristics of stay (origin of the patient: home, transfer from other hospital, emergency ward; category of care: medicine, surgery; intensive care unit (ICU) care: yes, no; major diagnosis groups based on ICD-10 chapters: infectious diseases, cancer, haematological diseases, endocrine, nutrition, and metabolism diseases, mental and behavioural disorders (i.e., mainly due to organic mental disorders and psychoactive substance use which are treated in acute/somatic care and not in psychiatry), diseases of the nervous system, sensory organ diseases, circulatory diseases, respiratory diseases, digestive diseases, dermatological diseases, musculoskeletal and connective diseases, genitourinary diseases and injury and poisoning; three selected acute medical conditions: stroke, acute myocardial infarction and sepsis) and hospital characteristics (academic hospital, other public hospital, private hospital)]. Stroke, acute myocardial infarction and sepsis were selected because they are leading causes of death globally and share the primary management requirement of rapid intervention. Previous works suggested that these three acute medical conditions were associated with fewer hospital admissions and/or increased mortality [28, 29].

### Statistical analysis

A difference-in-differences approach was used to determine whether mortality (primary outcome) during the COVID-19 period differed from the pre-COVID-19 period between patients with schizophrenia and patients without severe mental disorder. We employed a patient-level logistic regression model with three independent factors: patient group (with and without schizophrenia), period (pre-COVID-19 and COVID-19 periods) and an interaction term between patient group and period. By testing for an interaction between period and patient group, we assessed whether there was a difference in the change in mortality over time between the two groups. We completed the adjustment with ten unbalanced characteristics between the two groups during the pre-COVID-19 period (i.e., complementary health insurance, smoking addiction, alcohol addiction, substance addiction, Charlson comorbidity score, origin of the patient, category of care, ICU care, major diagnosis groups based on ICD-10 chapters and hospital characteristics) and significant interactions between these variables. The characteristics between the two groups were compared using standardized differences (SD) to identify meaningful differences before the pandemic. SD values greater than 0.10 were considered clinically significant [30] (Supplementary Table 1). We also performed stratified analyses on different medical conditions (ICD-10 chapters, category of care and acute medical conditions) to identify situations where the discrepancies were particularly sharpened.

A difference-in-differences approach was also used to determine whether the patient case mix (secondary outcome) during the COVID-19 period differed from the pre-COVID-19 period between the two groups. We employed a patient-level logistic regression model with three independent factors: patient group (with and without schizophrenia), period (pre-COVID-19 and COVID-19 periods) and an interaction term between patient group and period.

The results were expressed using odds ratios (ORs) and adjusted ORs (aORs) with their 95% confidence intervals (95% CIs). A significance threshold of *p* < 0.05 was used. All analyses were performed in SAS (version 9.4).

## Results

**Fig. 1:**
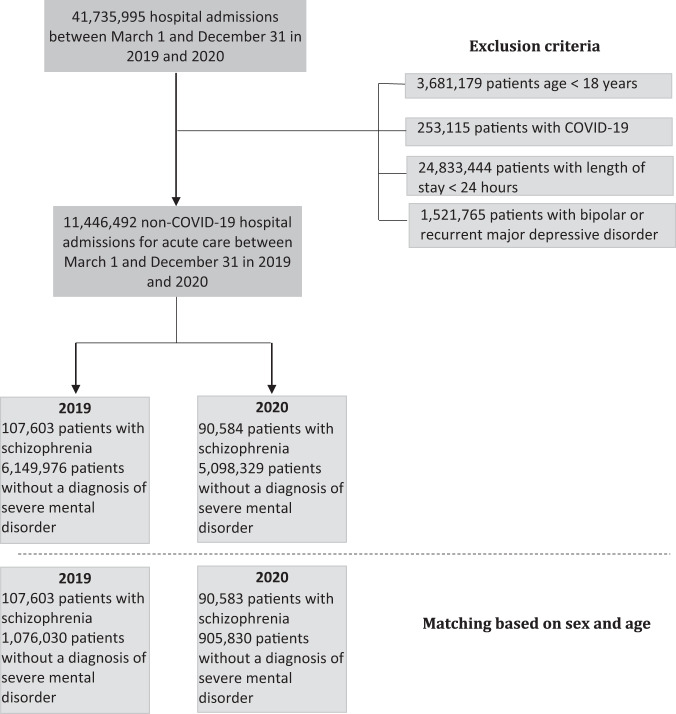
Flow chart.

Between the two periods, there was a 15.8% decrease in the number of admissions for patients with schizophrenia (107,603 in the pre-COVID-19 period and 90,584 in the COVID-19 period) and a 17.1% decrease in patients without a diagnosis of severe mental disorder (6,149,976 in the pre-COVID-19 period and 5,098,329 in the COVID-19 period) (flow chart, Fig. 1). A total of 198,186 patients with schizophrenia (46% female; age mean = 58.5 ± 19.5 years) were matched with 1,981,860 controls. The sociodemographic characteristics of patients are presented in Supplementary Table 2.

### Differential changes in mortality between patients with schizophrenia and matched controls

**Fig. 2:**
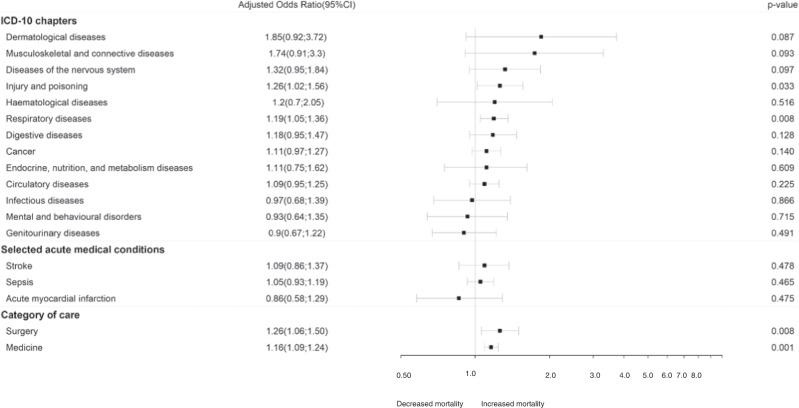
Forest plots of adjusted odds ratios for 90-day hospital mortality changes between the pre-COVID-19 and COVID-19 periods in patients with schizophrenia compared with patients without a diagnosis of severe mental disorder* stratified by ICD-10 chapters, selected acute medical conditions and category of care. * Matched for age and sex. Bold: *p* value < 0.05. 95% CI 95% confidence interval.

The 90-day hospital mortality in patients with schizophrenia increased significantly more than that of the matched controls without a diagnosis of severe mental disorder (+0.47% vs. +0.21%) without and after adjustment for the 10 unbalanced characteristics (aOR = 1.18; 95% CI = 1.11–1.25; *p* < 0.001). This increased mortality was significant for poisoning and injury (aOR = 1.26; 95% CI = 1.02–1.56; *p* = 0.033), respiratory diseases (aOR = 1.19; 95% CI = 1.05–1.36; *p* = 0.008) and in both surgery (aOR = 1.26; 95% CI = 1.06–1.50; *p* = 0.008) and medical care settings (aOR = 1.16; 95% CI, 1.09–1.24; *p* = 0.001) (Fig. 2).

The same increase was found for 30-day hospital mortality. Patients with schizophrenia increased significantly more than that of the matched controls without a diagnosis of severe mental disorder (+0.50% vs. +0.22%) without and after adjustment (aOR = 1.20; 95% CI = 1.13–1.27; *p* < 0.001). All the details are provided in Table 1.

**Table 1:** Change in mortality between the pre-COVID-19 and COVID-19 periods in patients with schizophrenia compared with patients without a diagnosis of severe mental disorder^a^.

	Patients with schizophrenia			Patients without a diagnosis of severe mental disorder^a^						
	Pre-COVID-19 period (*N* = 107,603)	COVID-19 period (*N* = 90,583)	Absolute difference (95% CI), percentage points	Pre-COVID-19 period (*N* = 1,076,030)	COVID-19 period (*N* = 905,830)	Absolute difference (95% CI), percentage points	Unadjusted odds ratio^b^ (95% CI)	*p* Value	Adjusted^c^ odds ratio (95% CI)	*p* Value
	*N* (%)	*N* (%)		*N* (%)	*N* (%)					
Principal outcome										
90-Day hospital mortality	2883 (2.68)	2857 (3.15)	0.47 (0.47; 0.48)	30334 (2.82)	27478 (3.03)	0.21 (0.21; 0.22)	1.10 (1.04; 1.16)	**<0.001**	1.18 (1.11; 1.25)	**<0.001**
Secondary outcome										
30-Day hospital mortality	2595 (2.41)	2634 (2.91)	0.50 (0.49; 0.50)	27846 (2.59)	25396 (2.80)	0.22 (0.21; 0.22)	1.12 (1.05; 1.18)	**<0.001**	1.20 (1.13; 1.27)	**<0.001**

Bold: *p* value < 0.05.

*95% CI* 95% confidence interval.

^a^Matched for age and sex.

^b^An odds ratio of 1.20 means that patients with schizophrenia have a 20% greater increase in the odds of death (COVID-19 period vs. pre-COVID-19 period) compared to matched controls (reference).

^c^Adjusted models included complementary health insurance (yes/no), smoking addiction (yes/no), alcohol addiction (yes/no), substance addiction (yes/no), Charlson comorbidity score (0/1–2/≥3), origin of the patient (home/transfer from other hospital/emergency ward), category of care (medicine/surgery), ICU care (yes/no), major diagnosis groups based on ICD-10 chapters and hospital characteristics (academic hospital/other public hospital/private hospital).

### Differential changes in patient case mix between patients with schizophrenia and matched controls

From the pre-COVID-19 period to the COVID-19 period, several significant differential changes in the case mix of admitted patients were found between patients with schizophrenia and patients without severe mental disorder. All the details are provided in Table 2.

**Table 2:** Change in patient case mix (admission-diagnosis distribution) between pre-COVID-19 and COVID-19 periods in patients with schizophrenia compared with patients without a diagnosis of severe mental disorder^a^.

	Patients with schizophrenia			Patients without a diagnosis of severe mental disorder^a^				
	Pre-COVID-19 period (*N* = 107,603)	COVID-19 period (*N* = 90,583)	Absolute difference (95% CI), percentage points	Pre-COVID-19 period (*N* = 1,076,030)	COVID-19 period (*N* = 905,830)	Absolute difference (95% CI), percentage points	Unadjusted odds ratio (95% CI)	*p* value
	*N* (%)	*N* (%)		*N* (%)	*N* (%)			
*Sociodemographic characteristics*								
Social deprivation								
Complementary health insurance^b^	10,359 (9.63)	13,798 (15.23)	5.61 (5.59; 5.62)	72,756 (6.76)	75,216 (8.30)	1.54 (1.53; 1.55)	1.35 (1.31; 1.39)	**<0.001**
*Comorbidities*								
Smoking addiction	17,460 (16.23)	15,430 (17.03)	0.81 (0.79; 0.82)	92,110 (8.56)	83,608 (9.23)	0.67 (0.66; 0.68)	0.98 (0.95; 1.00)	0.055
Alcohol addiction	19,755 (18.36)	17,363 (19.17)	0.81 (0.79; 0.83)	67,751 (6.30)	63,717 (7.03)	0.74 (0.73; 0.75)	0.94 (0.91;0.96)	**<0.001**
Substance addiction	32,831 (30.51)	28,487 (31.45)	0.94 (0.92; 0.96)	137,611 (12.79)	125,376 (13.84)	1.05 (1.04; 1.07)	0.95 (0.94; 0.97)	**<0.001**
Charlson score								
0	50,883 (47.29)	43,058 (47.53)	0.25 (0.22; 0.27)	546,600 (50.80)	448,294 (49.49)	−1.31 (−1.33; −1.29)	1.06 (1.05; 1.08)	**<0.001**
1–2	31,724 (29.48)	26,564 (29.33)	−0.16 (−0.18; −0.14)	288,896 (26.85)	247,251 (27.30)	0.45 (0.43; 0.47)	0.97 (0.95; 0.99)	**<0.001**
≥3	24,996 (23.23)	20,961 (23.14)	−0.09 (−0.11; −0.07)	240,534 (22.35)	210,285 (23.21)	0.86 (0.84; 0.88)	0.95 (0.93; 0.97)	**<0.001**
*Charlson comorbidities*								
Renal disease	9024 (8.39)	7357 (8.12)	−0.26 (−0.28; −0.25)	74,242 (6.90)	61,574 (6.80)	−0.10 (−0.11; −0.09)	0.98 (0.95; 1.01)	0.260
Liver mild disease	5296 (4.92)	4550 (5.02)	0.10 (0.09; 0.11)	37,925 (3.52)	33,476 (3.70)	0.17 (0.16; 0.18)	0.97 (0.93; 1.02)	0.206
Liver moderate/severe disease	1529 (1.42)	1256 (1.39)	−0.03 (−0.04; −0.03)	15,202 (1.41)	13,790 (1.52)	0.11 (0.10; 0.11)	0.90 (0.84; 0.98)	**0.012**
Peptic ulcer	1481 (1.38)	1048 (1.16)	−0.22 (−0.22; −0.21)	11,431 (1.06)	9715 (1.07)	0.01 (0.01; 0.01)	0.83 (0.76; 0.90)	**<0.001**
Chronic pulmonary disease	13,237 (12.30)	10835 (11.96)	−0.34 (−0.35; −0.33)	79680 (7.40)	66,293 (7.32)	−0.09 (−0.10; −0.08)	0.98 (0.95; 1.01)	0.192
Congestive heart failure	12,928 (12.01)	10,513 (11.61)	−0.41 (−0.42; −0.39)	120,522 (11.20)	101,070 (11.16)	−0.04 (−0.06; −0.03)	0.97 (0.94; 0.99)	**0.013**
Myocardial infarction	6431 (5.98)	5137 (5.67)	−0.31 (−0.32; −0.30)	73,645 (6.84)	62,975 (6.95)	0.11 (0.10; 0.12)	0.93 (0.90; 0.96)	**<0.001**
Peripheral vascular disease	5747 (5.34)	4796 (5.29)	−0.05 (−0.06; −0.04)	61,936 (5.76)	51,907 (5.73)	−0.03 (−0.04; −0.02)	1.00 (0.96; 1.04)	0.829
Cerebrovascular disease	7747 (7.20)	6622 (7.31)	0.11 (0.10; 0.12)	66,614 (6.19)	58,910 (6.50)	0.31 (0.30; 0.32)	0.96 (0.93; 0.99)	**0.045**
Dementia	11,362 (10.56)	9447 (10.43)	−0.13 (−0.14;−0.12)	37,404 (3.48)	29,968 (3.31)	−0.17 (−0.18; −0.16)	1.04 (1.00; 1.07)	**0.016**
Hemi-/paraplegia	4745 (4.41)	3980 (4.39)	−0.02 (−0.03; −0.01)	41,353 (3.84)	36,432 (4.02)	0.18 (0.17; 0.19)	0.95 (0.91; 0.99)	**0.029**
Rheumatic disease	1006 (0.93)	850 (0.94)	0.00 (−0.00; 0.01)	13,618 (1.27)	11,288 (1.25)	−0.02 (−0.02; −0.01)	1.02 (0.93; 1.12)	0.689
Metastatic solid tumour	4230 (3.93)	3815 (4.21)	0.28 (0.27; 0.29)	73,995 (6.88)	68,943 (7.61)	0.73 (0.72; 0.75)	0.96 (0.92; 1.01)	0.107
Malignancy	10,639 (9.89)	9352 (10.32)	0.44 (0.42; 0.45)	172,985 (16.08)	158,997 (17.55)	1.48 (1.46; 1.49)	0.94 (0.92; 0.97)	**<0.001**
Complicated diabetes	16,289 (15.14)	13,913 (15.36)	0.22 (0.21; 0.24)	132,237 (12.29)	111,137 (12.27)	−0.02 (−0.03; −0.01)	1.06 (1.02; 1.10)	0.008
Non-complicated diabetes	5628 (5.23)	4582 (5.06)	−0.17 (−0.18; −0.16)	45,455 (4.22)	35,039 (3.87)	−0.36 (−0.36; −0.35)	1.02 (0.99; 1.05)	0.145
AIDS/HIV	1041 (0.97)	932 (1.03)	0.06 (0.06; 0.07)	4616 (0.43)	3849 (0.42)	−0.00 (−0.01; −0.00)	1.07 (0.97; 1.19)	0.152
*Characteristics of stay*								
Origin of the patient								
Home	33,759 (31.37)	25,970 (28.67)	−2.70 (−2.72; −2.68)	611,087 (56.79)	493,175 (54.44)	−2.35 (−2.37; −2.32)	0.97 (0.95; 0.99)	**0.001**
Transfer from other hospital	10,000 (9.29)	8796 (9.71)	0.42 (0.40; 0.43)	62,774 (5.83)	55,693 (6.15)	0.31 (0.30; 0.32)	0.99 (0.96; 1.03)	0.655
Emergency ward	63,844 (59.33)	55,817 (61.62)	2.29 (2.27; 2.31)	402,169 (37.38)	356,962 (39.41)	2.03 (2.01; 2.05)	1.01 (0.99; 1.03)	0.313
Category of care								
Medicine	89,398 (83.08)	75,378 (83.21)	0.13 (0.12; 0.15)	3,994,747 (64.96)	3,321,189 (65.14)	0.19 (0.17; 0.21)	1.00	-
Surgery	18,205 (16.92)	15,206 (16.79)	−0.13 (−0.15; −0.12)	2,155,229 (35.04)	1,777,140 (34.86)	−0.19 (−0.21; −0.17)	1.01 (0.98; 1.03)	0.577
ICU care	12,424 (11.55)	10,461 (11.55)	0.00 (−0.01; 0.02)	132,577 (12.32)	119,327 (13.17)	0.85 (0.84; 0.87)	0.93 (0.90; 0.95)	**<0.001**
Major diagnosis groups based on ICD-10 chapters								
Infectious diseases	1823 (1.69)	1465 (1.62)	−0.08 (−0.08; −0.07)	20,784 (1.93)	15,906 (1.76)	−0.18 (−0.18; −0.17)	1.05 (0.98; 1.13)	0.178
Cancer	5853 (5.44)	5070 (5.60)	0.16 (0.15; 0.17)	125,689 (11.68)	114,538 (12.64)	0.96 (0.95; 0.98)	0.94 (0.91; 0.98)	**0.003**
Haematological diseases	1979 (1.84)	1844 (2.04)	0.20 (0.19; 0.20)	20,305 (1.89)	17,898 (1.98)	0.09 (0.08; 0.09)	1.06 (0.99; 1.13)	0.098
Endocrine, nutrition, and metabolism diseases	4606 (4.28)	3708 (4.09)	−0.19 (−0.20; −0.18)	45,867 (4.26)	34,725 (3.83)	−0.43 (−0.44; −0.42)	1.07 (1.02; 1.12)	**0.007**
Mental and behavioural disorders	22,530 (20.94)	19,869 (21.93)	1.00 (0.98; 1.01)	28,397 (2.64)	25,469 (2.81)	0.17 (0.17; 0.18)	0.99 (0.97; 1.02)	0.669
Diseases of the nervous system	4870 (4.53)	3925 (4.33)	−0.19 (−0.20; −0.18)	55,066 (5.12)	41,508 (4.58)	−0.54 (−0.54; −0.53)	1.07 (1.03; 1.12)	**0.002**
Sensory organ diseases	761 (0.71)	480 (0.53)	−0.18 (−0.18; −0.17)	18,252 (1.70)	12,795 (1.41)	−0.28 (−0.29; −0.28)	0.90 (0.80; 1.01)	0.080
Circulatory diseases	9315 (8.66)	7729 (8.53)	−0.12 (−0.14; −0.11)	157,895 (14.67)	138,702 (15.31)	0.64 (0.62; 0.65)	0.94 (0.91; 0.97)	**<0.001**
Respiratory diseases	9062 (8.42)	6960 (7.68)	−0.74 (−0.75; −0.73)	63,473 (5.90)	46,309 (5.11)	−0.79 (−0.80; −0.78)	1.05 (1.02; 1.09)	**<0.001**
Digestive diseases	8189 (7.61)	6777 (7.48)	−0.13 (−0.14; −0.12)	117,549 (10.92)	104,050 (11.49)	0.56 (0.55; 0.58)	0.93 (0.90; 0.96)	**<0.001**
Dermatological diseases	1419 (1.32)	1208 (1.33)	0.01 (0.01; 0.02)	16,747 (1.56)	13,139 (1.45)	−0.11 (−0.11; −0.10)	1.09 (1.00; 1.18)	**0.044**
Musculoskeletal and connective diseases	3918 (3.64)	2933 (3.24)	−0.40 (−0.41; −0.40)	102,002 (9.48)	78,002 (8.61)	−0.87 (−0.88; −0.86)	0.98 (0.94; 1.03)	0.530
Genitourinary diseases	4291 (3.99)	3745 (4.13)	0.15 (0.14; 0.16)	75,558 (7.02)	64,793 (7.15)	0.13 (0.12; 0.14)	1.02 (0.91; 1.07)	0.452
Injury and poisoning	14391 (13.37)	12,393 (13.68)	0.31 (0.29; 0.32)	105,939 (9.85)	93,663 (10.34)	0.49 (0.48; 0.51)	0.97 (0.95; 1.00)	0.044
Selected acute medical conditions								
Stroke	1496 (1.39)	1281 (1.41)	0.02 (0.02; 0.03)	19,624 (1.82)	18,560 (2.05)	0.23 (0.22; 0.23)	0.90 (0.84; 0.98)	**0.010**
Acute myocardial infarction	765 (0.71)	698 (0.77)	0.06 (0.06; 0.06)	17,026 (1.58)	16,066 (1.77)	0.19 (0.19; 0.20)	0.97 (0.87; 1.07)	0.514
Sepsis	5292 (4.92)	4543 (5.02)	0.10 (0.09; 0.11)	45,481 (4.23)	39,133 (4.32)	0.09 (0.08; 0.10)	1.00 (0.96; 1.04)	0.919
*Hospital characteristics*								
Academic	37,531 (34.88)	32,049 (35.38)	0.50 (0.48; 0.52)	297,716 (27.67)	255,349 (28.19)	0.52 (0.50; 0.54)	1.00 (0.98; 1.02)	0.694
Other public hospitals	60181 (55.93)	50,174 (55.39)	0.04 (0.02; 0.05)	487,606 (45.32)	408,375 (45.08)	−0.23 (−0.25; −0.21)	0.99 (0.97; 1.00)	0.191
Private	9891 (9.19)	8360 (9.23)	0.04 (0.02; 0.05)	290,708 (27.02)	242,106 (26.73)	−0.29 (−0.31; −0.27)	1.02 (0.99; 1.05)	0.229

Bold: *p* value < 0.05.

*95% CI* 95% confidence interval.

^a^Matched for age and sex.

^b^Having complementary health insurance indicates higher deprivation.

Patients with schizophrenia and multiple physical-health comorbidities were less hospitalized than matched controls (OR = 0.95; 95% CI = 0.93–0.97; *p* < 0.001), specifically for liver disease, peptic ulcer, congestive heart failure, myocardial infarction, cerebrovascular disease, hemi/paraplegia and malignancy.

Compared to matched controls, the admission rate was less increased in patients with schizophrenia for the following diseases: cancer (OR = 0.94; 95% CI = 0.91–0.98; *p* < 0.001), injury and poisoning (OR = 0.97; 95% CI = 0.95–1.00; *p* = 0.044), digestive diseases (OR = 0.93; 95% CI = 0.90–0.96; *p* < 0.001) and stroke (OR = 0.90; 95% CI = 0.84–0.98; *p* < 0.001). The admission rate was decreased for circulatory diseases in patients with schizophrenia, whereas it was increased in matched controls (OR = 0.94; 95% CI = 0.91–0.97; *p* < 0.001). The admission rate in the ICU was stable in patients with schizophrenia, whereas it was increased in matched controls (OR = 0.93; 95% CI = 0.90–0.95; *p* < 0.001).

In contrast, the admission rate decreased significantly less in patients with schizophrenia than that of the matched controls for respiratory (OR = 1.05; 95% CI = 1.02–1.09; *p* < 0.001), nutrition and metabolism (OR = 1.07; 95% CI = 1.02–1.12; *p* = 0.007), nervous system (OR = 1.07; 95% CI = 1.03–1.12; *p* = 0.002) and dermatological diseases (OR = 1.09; 95% CI = 1.00–1.18; *p* = 0.004).

## Discussion

In this nationwide population-based cohort study, we report a greater increase in 90-day hospital mortality among patients with schizophrenia than among patients without severe mental disorder. This increased mortality adds up to the previously described COVID-19-related excess mortality [13–17, 31]. We also report significant changes in patient case mix with a reduced admission in the ICU and for several somatic diseases for patients with schizophrenia compared to controls.

The 90-day hospital mortality in patients with schizophrenia increased significantly more than that of the patients without a diagnosis of severe mental disorder. This increased mortality was maintained after adjustment for a large range of sociodemographic, clinical and stay characteristics, suggesting that the quality and safety of care given to patients (and not their social or medical conditions at admission) was a true contributor to the increase in the odds of mortality in schizophrenia. Previous works suggest that standard care provided to non-COVID-19 patients has been affected by overwhelming workload and resource prioritization, potentially leading to safety issues with adverse events occurrence and failure to rescue (i.e., failure to prevent inpatient deterioration and death) [1, 32]. Several factors may explain why patients with schizophrenia have been more affected by adverse events and failure to rescue compared with controls. Indeed, the management of patients with schizophrenia is complex and requires a high level of skills and staff involvement. Uncontrolled schizophrenia decreases the ability of the patient to adequately report her or his symptoms and needs. The concept of diagnostic overshadowing is described as inadequate or delayed treatment due to misattribution of physical symptoms to mental illness [33–35]. In addition, diagnostic overshadowing may be worsened by oversedation due to excessive doses of psychotropics to control agitation [27] or the difficulty of combining psychotropic drugs with perioperative medications [36]. Previous works also reported an increased risk of postoperative complications, including respiratory failure, sepsis, deep venous thrombosis, pulmonary embolism, paralytic ileus, stroke, and postoperative delirium, compared with people without mental disorders [37]. During times of surge from COVID-19 cases, professionals may have been less conducive to the timely recognition and effective management of these complications. Although the ICD-10 code U071* has been shown to have a high sensitivity and specificity for detecting COVID-19 hospital stays [22], we cannot exclude that the observed increase in mortality for respiratory diseases in patients with schizophrenia can be explained by undiagnosed/unreported COVID-19. Patients with schizophrenia should thus be targeted as a high-risk population for complications and risk of failure and should benefit from more intensive monitoring, early warning score systems and rapid response teams, taking into account the specificities of patients with schizophrenia (e.g., oversedation signs, symptoms attributable to mental illness or psychotropic drugs, respiratory depression) [38]. Staff skills and training must also be reinforced, as physician and nurse staff (including those working in the ICU [39]) report insufficient training to address the complex needs of patients with schizophrenia.

Changes in patient case mix suggest disparities in access to care between patients with and without schizophrenia during the pandemic. Patients with schizophrenia were less admitted for several somatic diseases (especially cancer, circulatory and digestive diseases and stroke), accentuating the differences already found during the pre-COVID-19 period (Supplementary Table 1). Our findings also suggest less access to care for patients with schizophrenia and multiple physical health comorbidities. Access to and quality of health care for somatic diseases are major concerns in patients with schizophrenia [6, 9, 19]. Previous works reported that patients with schizophrenia received lower access and quality treatment for somatic diseases compared with patients without mental disorders [10, 12, 40, 41]. These results may conceal excess prehospital mortality and need to be studied further to measure the exact impact of the pandemic on patients with schizophrenia. The ICU admission rate was stable in patients with schizophrenia, whereas it was increased in patients without severe mental disorders, suggesting ICU triage for patients with schizophrenia. ICU triage was previously reported for COVID-19 patients with schizophrenia [15]. To date, few studies have investigated ICU access for patients with schizophrenia and reasons for triage. The ICU may be less prone to admit a patient with schizophrenia due to potential behavioural/aggressive disturbances and their inability to monitor them properly. Future work should determine how health inequities are mitigated during the ICU admission process for patients with schizophrenia.

In contrast, the admission rate decreased significantly less in patients with schizophrenia for respiratory, nutrition and metabolism, nervous system and dermatological diseases, suggesting less stabilized chronic conditions and more severe presentations. Previous work reported the impact of dermatological, respiratory, endocrine and neurological diseases on functioning levels and disability in schizophrenia, [42] which may have exacerbated illnesses especially during periods of reduced psychiatric care delivery [43]. These results need to be studied further to understand the underlying mechanisms and to develop preventive interventions.

### Limitations and perspectives

The impact of COVID-19 on non-COVID-19 mortality and hospital admissions may depend on health care organization and public management strategies. Our results may not be extrapolated to other countries and should thus be replicated. The impact of context (e.g., social distancing, isolation, lockdown) could also be explored and it would be interesting to determine whether it has affected patients with schizophrenia more than control patients. A weakness of medico-administrative databases is the miscoding of diagnoses during hospital stays that can underestimate patients’ comorbidities and severity at admission. Smoking addiction (Yes/No) and alcohol addiction (Yes/No) are crude binary classifications that cannot adequately measure these important confounding influences. Administrative data are known to exhibit high specificity but low sensitivity for capturing adverse events; thus, these events are often underreported [44] and were not analyzed in our study. These issues may be accentuated in schizophrenia given the phenomenon of diagnostic overshadowing [33–35]. In addition, several data, such as social isolation, race/ethnicity and treatments, were not available in our database and are known to influence health outcomes. Our work describes only patients who were admitted into acute care hospital, and we have no information on care and death occurring outside the hospital setting. These deaths might be differentially distributed by study group and time period with likely higher rates of out-of-hospital deaths among people with schizophrenia. Future work should study the impact of COVID-19 on non-COVID-19 out-of-hospital mortality. In our analysis, we did not distinguish index hospital stays from hospital readmissions. Future studies should determine whether increased mortality was related to early mortality during the index stay or later during hospital readmission. Some patients with schizophrenia may not have been identified in our study because we only selected patients diagnosed in acute and psychiatric hospitals. However, this problem should be minimized, as this study covered 5 years of hospitalization, and previous studies have shown that 83% of patients with schizophrenia are followed by public mental health hospitals in France [45]. In addition, since the PMSI database collects nationwide data, admissions of subjects diagnosed with schizophrenia in France and admitted to hospitals outside of France would have been missed. Finally, it would be interesting to determine whether our findings are specific for schizophrenia or whether just any patients with mental disorders could be affected in the same way.

## Conclusion

Our findings suggest a greater deterioration in access to, effectiveness and safety of non-COVID-19 acute care in patients with schizophrenia compared to patients without severe mental disorder during the COVID-19 pandemic. Since the beginning of the COVID-19 pandemic, physical health care in patients with schizophrenia more than ever represents a public health and ethical priority.

## Supplementary information

Supplementary material
