# Supplementary material for: Impact of the COVID-19 pandemic on non-COVID-19 hospital mortality in patients with schizophrenia: a nationwide population-based cohort study
Source: Mol Psychiatry. 2022 Oct 7;27(12):5186–94. doi: 10.1038/s41380-022-01803-4 (PMC9542474; doi:10.1038/s41380-022-01803-4)
Supplement: Supplementary file 1 — Supplementary material [file 41380_2022_1803_MOESM1_ESM.docx]

**Supplementary Table 1.** Difference in patient case mix (admission diagnosis distribution) during the pre-COVID-19 period between patients with schizophrenia and patients without a diagnosis of severe mental disorder*.

| Pre-COVID 19 period | Patients with schizophrenia  (N=107,603)  N (%) | Patients without a diagnosis of severe mental disorder* (N=1,076,030)  N (%) | Standardized difference |
| --- | --- | --- | --- |
| Sociodemographic characteristics |  |  |  |
| Social deprivation |  |  |  |
| Complementary health insurance | 10359 (9.63) | 72756 (6.76) | **0.10** |
| Comorbidities |  |  |  |
| Smoking addiction | 17460 (16.23) | 92110 (8.56) | **0.23** |
| Alcohol addiction | 19755 (18.36) | 67751 (6.30) | **0.37** |
| Substance addiction | 32831 (30.51) | 137611 (12.79) | **0.44** |
| Charlson score |  |  |  |
| 0 | 50883 (47.29) | 546600 (50.80) | -0.07 |
| 1-2 | 31724 (29.48) | 288896 (26.85) | 0.06 |
| ≥3 | 24996 (23.23) | 240534 (22.35) | 0.02 |
| Charlson comorbidities |  |  |  |
| Renal disease | 9024 (8.39) | 74242 (6.90) | 0.06 |
| Liver mild disease | 5296 (4.92) | 37925 (3.52) | 0.07 |
| Liver moderate/severe disease | 1529 (1.42) | 15202 (1.41) | 0.00 |
| Peptic ulcer | 1481 (1.38) | 11431 (1.06) | 0.03 |
| Chronic pulmonary disease | 13237 (12.30) | 79680 (7.40) | 0.16 |
| Congestive heart failure | 12928 (12.01) | 120522 (11.20) | 0.03 |
| Myocardial infarction | 6431 (5.98) | 73645 (6.84) | -0.04 |
| Peripheral vascular disease | 5747 (5.34) | 61936 (5.76) | 0.04 |
| Cerebrovascular disease | 7747 (7.20) | 66614 (6.19) | 0.04 |
| Dementia | 11362 (10.56) | 37404 (3.48) | **0.28** |
| Hemi/Paraplegia | 4745 (4.41) | 41353 (3.84) | 0.03 |
| Rhumatic disease | 1006 (0.93) | 13618 (1.27) | -0.03 |
| Metastatic solid tumour | 4230 (3.93) | 73995 (6.88) | **-0.13** |
| Malignancy | 10639 (9.89) | 172985 (16.08) | **-0.18** |
| Complicated diabetes | 16289 (15.14) | 132237 (12.29) | 0.05 |
| Non complicated diabetes | 5628 (5.23) | 45455 (4.22) | 0.08 |
| AIDS/HIV | 1041 (0.97) | 4616 (0.43) | 0.06 |
| Characteristics of stay |  |  |  |
| Origin of the patient |  |  |  |
| Home | 33759 (31.37) | 611087 (56.79) | **-0.53** |
| Transfer from other  Hospital | 10000 (9.29) | 62774 (5.83) | **0.13** |
| Emergency ward | 63844 (59.33) | 402169 (37.38) | **0.45** |
| Category of care |  |  |  |
| Medicine | 89398 (83.08) | 3994747 (64.96) | - |
| Surgery | 18205 (16.92) | 2155229 (35.04) | **-0.46** |
| ICU care | 12424 (11.55) | 132577 (12.32) | -0.02 |
| Major diagnosis groups based on ICD-10 chapters |  |  |  |
| Infectious diseases | 1823 (1.69) | 20784 (1.93) | -0.02 |
| Cancer | 5853 (5.44) | 125689 (11.68) | **-0.22** |
| Haematological diseases | 1979 (1.84) | 20305 (1.89) | -0.00 |
| Endocrine, nutrition, and metabolism diseases | 4606 (4.28) | 45867 (4.26) | 0.00 |
| Mental and behavioural disorders | 22530 (20.94) | 28397 (2.64) | **0.59** |
| Diseases of the nervous system | 4870 (4.53) | 55066 (5.12) | -0.03 |
| Sensory organ diseases | 761 (0.71) | 18252 (1.70) | -0.09 |
| Circulatory diseases | 9315 (8.66) | 157895 (14.67) | **-0.19** |
| Respiratory diseases | 9062 (8.42) | 63473 (5.90) | 0.10 |
| Digestive diseases | 8189 (7.61) | 117549 (10.92) | **-0.11** |
| Dermatological diseases | 1419 (1.32) | 16747 (1.56) | 0.10 |
| Musculoskeletal and connective diseases | 3918 (3.64) | 102002 (9.48) | **-0.24** |
| Genitourinary diseases | 4291 (3.99) | 75558 (7.02) | **-0.13** |
| Injury and poisoning | 14391 (13.37) | 105939 (9.85) | **0.11** |
| Selected acute medical conditions |  |  |  |
| Stroke | 1496 (1.39) | 19624 (1.82) | -0.03 |
| Acute myocardial infarction | 765 (0.71) | 17026 (1.58) | -0.08 |
| Sepsis | 5292 (4.92) | 45481 (4.23) | 0.03 |
| Hospital characteristics |  |  |  |
| Academic | 37531 (34.88) | 297716 (27.67) | **0.19** |
| Other public hospital | 60181 (55.93) | 487606 (45.32) | **0.23** |
| Private | 9891 (9.19) | 290708 (27.02) | **-0.48** |

* Matched for age and sex.

Bold: standardized difference>0.10, considered clinically significant.

**Supplementary Table 2.** Sociodemographic characteristics of patients with schizophrenia and patients without a diagnosis of severe mental disorder*.

|  | Patients with schizophrenia | | | Patients without a diagnosis of severe mental disorder* | | |
| --- | --- | --- | --- | --- | --- | --- |
|  | Pre-COVID-19 period  (N=107,603)  N (%) | COVID-19 period (N=90,583)  N (%) | Absolute difference (95% CI),  percentage points | Pre-COVID-19 period  (N=1,076,030)  N (%) | COVID-19 period  (N=905,830)  N (%) | Absolute difference (95% CI),  percentage points |
| Sociodemographic characteristics |  |  |  |  |  |  |
| Age classes, year |  |  |  |  |  |  |
| 18-24 | 4380 (4.07) | 3622 (4.00) | -0.07 (-0.08; -0.06) | 43800 (4.07) | 36220 (4.00) | -0.07 (-0.08; -0.06) |
| 25-34 | 10152 (9.43) | 8874 (9.80) | 0.36 (0.35; 0.37) | 101520 (9.43) | 88740 (9.80) | 0.36 (0.35; 0.37) |
| 35-44 | 13058 (12.14) | 11173 (12.33) | 0.20 (0.18; 0.21) | 130580 (12.14) | 111730 (12.33) | 0.20 (0.18; 0.21) |
| 45-54 | 17993 (16.72) | 14917 (16.47) | -0.25 (-0.27; -0.24) | 179930 (16.72) | 149170 (16.47) | -0.25 (-0.27;-0.24) |
| 55-64 | 18412 (17.11) | 15688 (17.32) | 0.21 (0.19; 0.22) | 184120 (17.11) | 156880 (17.32) | 0.21 (0.19; 0.22) |
| 65-74 | 18032 (16.76) | 15450 (17.06) | 0.30 (0.28; 0.31) | 180320 (16.76) | 154500 (17.06) | 0.30 (0.28; 0.31) |
| 75-84 | 13934 (12.95) | 11750 (12.97) | 0.02 (0.01; 0.04) | 139340 (12.95) | 117500 (12.97) | 0.02 (0.01; 0.04) |
| 85-94 | 10566 (9.82) | 8221 (9.08) | -0.74 (-0.76; -0.73) | 105660 (9.82) | 82210 (9.08) | -0.74 (-0.76; -0.73) |
| ≥94 | 1076 (1.00) | 888 (0.98) | -0.02 (-0.02; -0.02) | 10760 (1.00) | 8880 (0.98) | -0.02 (-0.02; -0.02) |
| Sex |  |  |  |  |  |  |
| Male | 57063 (53.03) | 48697 (53.76) | 0.73 (0.71 ;0.75) | 570630 (53.03) | 486970 (53.76) | 0.77 (0.74; 0.79) |
| Female | 50540 (46.97) | 41886 (46.24) | -0.73 (-0.75; -0.71) | 505400 (46.97) | 418860 (46.24) | -0.77 (-0.79; -0.74) |

*Matched for age and sex.

95% CI: 95% confidence interval.
